# Cerebrovascular Risk Profiles in a Saudi Arabian Cohort of Young Stroke Patients

**DOI:** 10.3389/fneur.2021.736818

**Published:** 2021-11-12

**Authors:** Marwa Ahmed Eltemamy, Arturo Tamayo, Eyad Altarsha, Annahita Sedghi, Lars-Peder Pallesen, Jessica Barlinn, Volker Puetz, Ben Min-Woo Illigens, Kristian Barlinn, Timo Siepmann

**Affiliations:** ^1^Department of Stroke Medicine, Fairfield General Hospital, Manchester, United Kingdom; ^2^Division of Health Care Sciences, Center for Clinical Research and Management Education, Dresden International University, Dresden, Germany; ^3^Department of Neurology, King Abdullah Medical City, Mecca, Saudi Arabia; ^4^Winnipeg Regional Health Authority (WRHA), Department of Medicine, Section of Neurology, The Max Rady Faculty of Health Sciences, Brandon Regional Health Centre, University of Manitoba, Winnipeg, MB, Canada; ^5^Department of Neurology, Carl Gustav Carus University Hospital, Technische Universität Dresden, Dresden, Germany; ^6^Department of Neurology, Beth Israel Deaconess Medical Center, Harvard Medical School, Boston, MA, United States

**Keywords:** stroke, ischemic stroke, stroke in young, Saudi Arabia, risk factors, juvenile, infarction, ischemia

## Abstract

**Background:** The constantly increasing incidence of stroke in younger individuals substantiates an urgent need for research to elucidate underlying risk factors and etiologies. Heretofore, the vast majority of studies on stroke in the young have been carried out in European and North American regions. We aimed to characterize cerebrovascular risk profiles in a Saudi Arabic cohort of consecutive young stroke patients.

**Methods:** We retrospectively analyzed data from consecutive ischemic stroke patients aged 15 to 49 years who underwent detailed cardiocerebrovascular evaluation at a tertiary stroke care center in Makkah, Saudi Arabia. Distributions of risk factors and stroke etiologies were assessed in the entire cohort and in two strata of very young (15–40 years) and young to middle-aged patients (41–49) to account for variability in suggested age cutoffs.

**Results:** In the entire cohort [*n* = 63, ages 44 (34–47) median, interquartile range], dyslipidemia (71.4%) and small vessel occlusion (31.7%) displayed highest prevalence followed by diabetes (52.4%) and cardioembolism (19%). In very young patients, cardioembolism was the most prevalent etiology (27.3%). Risk profiles were similar between both age strata except for a higher prevalence of diabetes among the older cohort (31.8 vs. 63.4%, *p* = 0.01). Logistic regression identified diabetes as strongest predictor for association to the older strata (odds ratio = 4.2, 95% confidence interval = 1.2–14.1, *p* = 0.02).

**Conclusion:** Cerebrovascular risk profiles and stroke etiologies in our cohort of young stroke patients differ from those of previous cohorts, suggesting the need for tailored prevention strategies that take into account local epidemiological data on cerebrovascular health.

## Introduction

Stroke is the third leading cause of death and a major cause of disability in survivors. More than 65% of stroke-related deaths occur in developing countries (1, 2). While stroke in the elder population has been studied extensively, the distribution of risk factors and stroke etiologies in the young has not been fully elucidated to date. Moreover, there is no clear age cut-off to define young people. However, several studies defined juvenile stroke as stroke that occurs before the age of 50 years. Stroke in individuals younger than 18 years is frequently referred to as pediatric stroke, an entity that is equally understudied (3–8). Approximately 10% of ischemic strokes occur in this age group (9, 10). The repercussions seen in juvenile stroke are socioeconomically higher than in the elderly because of its devastating disability during highly productive years. Recently, several studies have shown a rise in the incidence of ischemic stroke at younger ages since the 1980s with the concurrent decline of incidence at older ages (11–16).

Various studies assessed etiologies and risk factors of stroke in young adults from different countries, the majority of which located in North America or Europe (3, 17–30). These studies showed substantial geographic and ethnic differences of cerebrovascular risk profiles and distribution of etiologies of stroke in the young. In the Kingdom of Saudi Arabia, acute stroke is a frequent disorder with an incidence of 30 to 40 per 1,00,000 per year and a prevalence of 186 per 1,00,000. However, the local epidemiology and clinical presentation of stroke in the young are unknown to date (30, 31). Defining patterns of modifiable cerebrovascular risk factors and stroke etiologies in the population of young individuals with acute stroke in this region might help develop tailored preventive strategies to improve cerebrovascular health. Therefore, we sought to characterize cerebrovascular risk profiles in a Saudi Arabic cohort of consecutive juvenile stroke patients.

## Materials and Methods

### Design, Population, and Protocol

We carried out a retrospective analysis including data on consecutive acute ischemic stroke patients from the electronic medical records data bank of the tertiary stroke center at King Abdullah Medical City (KAMC) in Makkah, Saudi Arabia. Patients aged 15 to 49 years with ischemic stroke at the time of presentation from January 2012 to December 2018 were included. We explicitly chose to include patients younger than 18 years but older than 14 years that would be considered pediatric stroke rather than juvenile stroke to allow an analysis in two age strata of young stroke patients. Thus, we tolerated an overlap between the definition of our very young stroke cohort and common agreement on the definition of pediatric stroke. The main reason for this approach was the arbitrary nature of commonly accepted age cutoffs and our goal to test new age strata that have not been used before. However, this approach later turned out to remain anecdotal as all of our patients were, in fact, older than 18 years. Diagnosis of acute ischemic stroke was done by a stroke neurologist and was confirmed by a neuroradiologist based on cerebral imaging [computed tomography (CT) or magnetic resonance imaging (MRI)]. Patients with hemorrhagic stroke, cerebral venous thrombosis, iatrogenic stroke secondary to a procedure or operation, or transient ischemic attack were excluded. The Trial of Org 10172 in Acute Stroke Treatment criteria was applied to categorize stroke etiologies (32). To assess age-related trends, our study population was dichotomized according to their age into a very young cohort who are 40 years or younger and a young to middle-aged cohort who are 41 to 49 years old. The most common risk factors were explored including demographics, smoking, alcohol, obesity, illicit drugs, family history, hypertension, diabetes mellitus, dyslipidemia, and cardiovascular disease. They were matched to definitions of the Helsinki Young Stroke Registry, which is a large study of ischemic stroke in young adults (3). Thrombophilia screen (including protein C, protein S, antithrombin III, prothrombin mutation gene, and factor V Leiden mutation) and autoimmune profile (including antinuclear antibodies, anti–double-stranded deoxyribonucleic acid antibodies, antineutrophil cytoplasmic antibodies, anticardiolipin antibodies, and lupus anticoagulants), which are routinely requested to young people admitted with stroke, were also included in our analysis.

Cerebrovascular risk profiles and etiologies were further assessed using results of cerebral imaging (cranial CT or MRI) and vessel imaging (CT angiography to head and neck or magnetic resonance time-of-flight angiography of brain vessels combined bilateral carotid Doppler). Accordingly, the vascular territory was classified as anterior circulation, posterior circulation, or both, and stroke location was categorized as the cerebral, cerebellar, brain stem, or multiple. To complement cerebrovascular phenotyping, cardiac tests were undertaken, including transthoracic echocardiography as well as Holter monitoring for 48 h. Transesophageal echocardiography has not yet been established as institutional standard at the time of study commencement and was therefore performed at the discretion of the treating stroke physician.

### Ethics

The study was conducted according to the guidelines of the Declaration of Helsinki and approved by the institutional review board of KAMC (IRB no. 18-485, date of approval April 24, 2019).

### Statistical Analysis

Analysis was performed using SPSS (IBM SPSS Statistics for Windows, version 22.0, released 2013; IBM Corp., Armonk, NY). Available case analysis was carried out. Data are presented as absolute and relative frequencies or median and interquartile range (IQR) according to type and distribution. Age strata, groups of male and female patients, and Saudi and non-Saudi patient groups were compared using Pearson χ^2^ for categorical variables. A two-sided *p* < 0.05 was considered significant. Fisher exact test was used whenever needed to confirm results. Additional logistic regression analysis was performed to identify potential predictors for association to either age group.

## Results

### Study Population

From January 2012 to December 2018, 63 patients younger than 50 years were admitted to KAMC with ischemic stroke. Of these, 43 patients (68.3%) were males, and 20 patients (31.8%) were females. The median age of patients in this study was 44 years (*IQR* = 34–47 years). There was no difference in gender ratio between the two age groups (males/females: very young: 8/17 vs. 12/26; *p* = 1.0). Forty-six patients (73.0%) of the population were Saudi, whereas 17 patients (27.0%) were from other nationalities (from Pakistan, Bangladesh, and Yemen). The very young strata of stroke patients aged 15 to 40 years consisted of 25 patients (39.7%), whereas the young to middle-aged strata including stroke patients aged 41 to 49 years comprised 38 patients (60.3%). Etiology results according to Toast classification provided a complete data set.

### Cerebrovascular Risk Profiles

**Figure 1:**
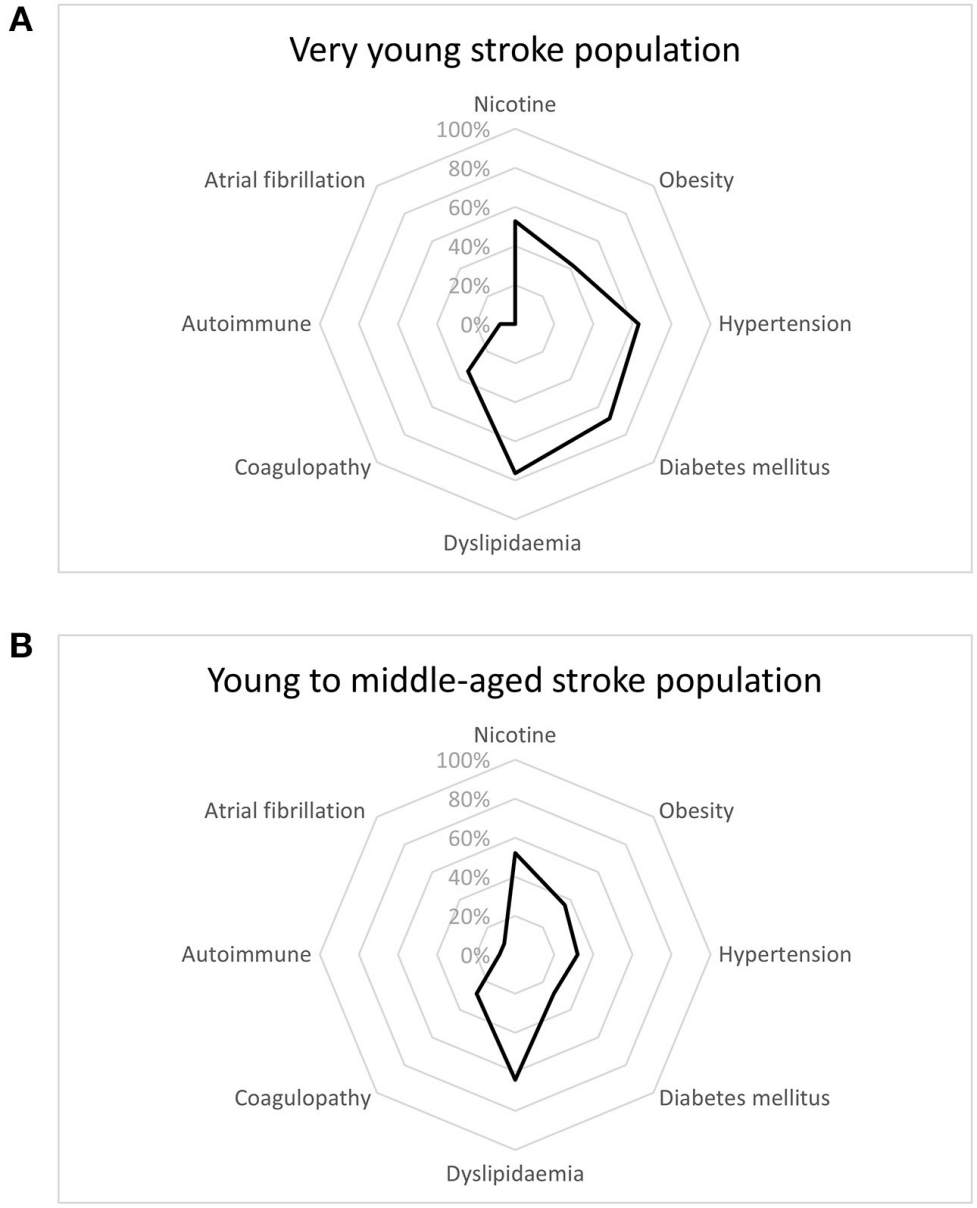
Distribution of cerebrovascular risk factors in the very young stroke population **(A)** as compared to the young **(B)** stroke population.

Dyslipidemia was the risk factor most frequently seen for stroke in our cohort (71.4%), followed by smoking (52.4%), diabetes mellitus (52.4%) and hypertension (50.8%). The most prevalent risk factor in the very young cohort was dyslipidemia (64%), followed by smoking (52%), whereas in young to middle-aged cohort, dyslipidemia was the most frequent (76.3%), with diabetes mellitus being the second most frequent risk factor (68.4%). In male patients, dyslipidemia was the most frequently found risk factor (79%), followed by smoking (74.4%). Among female patients, both dyslipidemia and obesity displayed highest frequencies (both 55%), followed by diabetes mellitus (45%). Comparing the two-age strata revealed a higher prevalence of diabetes (68.4 vs. 28%, *p* = 0.001) in the young to middle-aged cohort. Patterns of risk factors among age groups are depicted in Figure 1. On analysis of risk factors by gender, cardiovascular disease was more often present in males (34.9 vs. 5%, *p* = 0.01) than in females. Risk factor profiles are detailed in Table 1. Logistic regression analysis identified diabetes mellitus as predictor for association to either age group, with the older patients suffering more frequently from diabetes [odds ratio (*OR*) = 4.2, 95% confidence interval (*CI*) = 1.2–14.1, *p* = 0.02]. Distribution of cerebrovascular risk factors in the very young stroke population compared to the young to middle-aged stroke population is depicted in Figure 1.

**Table 1:** Cerebrovascular risk profiles (*n* = 63).

		**Age in years (no. [%])**			**Gender (no. [%])**			**Nationality (no. [%])**		
	Total^*^	**15–40**	**41–49**	***p***	**Male**	**Female**	***p***	**Saudi**	**Other**	***p***
	**(no. [%])**	**(25, 39.7%)**	**(38, 60.3%)**		**(43, 68.2%)**	**(20, 31.8%)**		**(46, 73.1%)**	**(17, 26.9%)**	
Smoking	33 (52.4%)	13 (52%)	20 (52.6%)	0.96	32 (74.4%)	1 (5%)	**0.001**	23 (50%)	10 (58.8%)	0.53
Obesity	25 (38%)	9 (36.4%)	16 (39%)	0.62	14 (31%)	11 (55%)	0.09	18 (36.1%)	7 (43.7%)	0.88
Hypertension	32 (50.8%)	8 (32%)	24 (63.2%)	**0.01**	24 (55.8%)	8 (40%)	0.24	25 (54.3%)	7 (41.2%)	0.35
Diabetes	33 (52.4%)	7 (28%)	26 (68.4%)	**0.001**	24 (55.8%)	9 (45%)	0.42	23 (50%)	10 (58.8%)	0.53
Dyslipidemia	45 (71.4%)	16 (64%)	29 (76.3%)	0.62	34 (79%)	11 (55%)	0.06	34 (73.9%)	11 (64.7%)	0.47

*no., number; Saudi, Saudi Arabian citizen*.

* *Numbers derived from available case analysis. Bold indicates statistical significance*.

### Juvenile Stroke Etiology

The most frequent stroke etiology in our study population was small vessel occlusion (31.7%), followed by cardioembolism (19%) and large-artery atherosclerosis (12.7%). In the very young strata, cardioembolism and small vessel occlusion were more prevalent (each 24%), whereas in the young to middle-aged strata, small vessel disease was more frequent (36.8%) (Table 2). Cause of cardioembolic strokes comprised intracardiac thrombus (*n* = 6), ischemic cardiomyopathy (*n* = 2), dilated cardiomyopathy (*n* = 1), atrial fibrillation (*n* = 2), and patent foramen ovale (*n* = 1). Eleven young stroke patients (17.5%) were categorized as other determined cause of stroke. Diagnostic workup revealed artery dissection (*n* = 4), rare arterial diseases (*n* = 3), autoimmune vasculitis (*n* = 1), hypoperfusion with bilateral watershed infarcts (*n* = 1), decreased protein S levels (*n* = 1), and antiphospholipid syndrome with positive test of anticardiolipin antibodies (*n* = 1). In the patient with antiphospholipid syndrome, test of anticardiolipin antibodies was still positive at repeated testing 3 months later. Twelve patients (19%) had cryptogenic stroke. The prevalence of stroke etiologies did not differ between males and females. On logistic regression, stroke etiology displayed no predictive association with either of the two age strata (very young and young to middle-aged: *OR* = 1.2, 95% CI = 0.7–1.8, *p* = 0.5).

**Table 2:** Distribution of stroke etiologies (*n* = 63).

		**Age in years (no. [%])**			**Gender (no. [%])**			**Nationality (no. [%])**		
	Total^*^	**15–40**	**41–49**	***p***	**Male**	**Female**	***p***	**Saudi**	**Other**	***p***
	**(no. [%])**	**(25, 39.7%)**	**(38, 60.3%)**		**(43, 68.2%)**	**(20, 31.8%)**		**(46, 73.1%)**	**(17, 26.9%)**	
Small vessel occlusion	20 (31.7%)	6 (24%)	14 (36.8%)	0.28	15 (34.9%)	5 (25%)	0.43	14 (30.4%)	6 (35.3%)	0.71
Cardioembolism	12 (19%)	6 (24%)	6 (15.8%)	0.42	10 (23.3%)	2 (10%)	0.3	12 (26%)	0 (0%)	**0.03**
Atherothrombotic	8 (12.7%)	4 (16%)	4 (10.5%)	0.7	5 (11.6%)	3 (15%)	0.7	5 (10.9%)	3 (17.6%)	0.67
Other	11 (17.5%)	5 (20%)	6 (15.8%)	0.74	5 (11.6%)	6 (30%)	0.09	9 (19.6%)	2 (11.8%)	0.71
Cryptogenic	12 (19%)	4 (16%)	8 (21%)	0.75	8 (18.6%)	4 (20%)	1	6 (13%)	6 (35.3%)	0.46
Protein C deficiency	0	0	0		0	0		0	0	
Protein S deficiency	5 (7.9%)	3 (12%)	2 (5.3%)	0.74	3 (7%)	2 (10%)	0.46	4 (8.7%)	1 (5.9%)	0.84
AT III deficiency	3 (4.8%)	1 (4%)	2 (5.3%)	0.45	2 (4.7%)	1 (5%)	0.96	3 (6.5%)	0	0.9
Prothrombin mutation	0	0	0	0.34	0	0	0.73	0	0	0.51
Lupus anticoagulant	0	0	0	0.1	0	0	0.4	0	0	0.1
Anticardiolipin ABs	4 (6.3%)	0	4 (10.5%)	0.25	2 (4.7%)	2 (10%)	0.68	4 (8.7%)	0	0.77
Factor V Leiden	0	0	0	0.3	0	0	0.1	0	0	0.95
Homocysteine	8 (12.7%)	3 (12%)	5 (13.2%)	0.28	5 (11.6%)	3 (15%)	0.86	6 (13%)	2 (11.8%)	0.86
ANA	3 (4.8%)	2 (8%)	1 (2.6%)	0.48	1 (2.3%)	2 (10%)	0.23	2 (4.3%)	1 (5.9%)	0.54
ANCA	1 (1.6%)	0	1 (2.6%)	0.92	0	1 (5%)	0.52	1 (2.2%)	0	0.35
Low EF	16 (25.4%)	2 (8%)	14 (36.8%)	**0.018**	15 (34.9%)	1 (5%)	**0.01**	12 (26%)	4 (23.5%)	0.76
Atrial fibrillation	2 (3.2%)	2 (8%)	0	0.31	1 (2.3%)	1 (5%)	0.57	2 (4.3%)	0	0.83
LV thrombus	5 (7.9%)	1 (4%)	4 (10.5%)	0.54	4 (9.3%)	1 (5%)	0.37	5 (10.9%)	0	0.5

*ABs, antibodies; ANA, antinuclear antibodies; ANCA, antineutrophil cytoplasmic antibodies; AT, antithrombin; EF, ejection fraction; LF, left ventricular; no., number*.

* *Numbers derived from available case analysis. Bold indicates statistical significance*.

### Vascular Territories

Anterior circulation stroke was noted in 44 patients (69.8 %), whereas 11 patients (17.5%) of our study population had posterior circulation stroke. Eight patients (12.7%) had a stroke in both vascular territories (Table 3). There was no difference between males and females or age strata regarding the vascular territory involved.

**Table 3:** Anatomical distribution of ischemic stroke by circulation territory (*n* = 63).

		**Age in years (no. [%])**			**Gender (no. [%])**			**Nationality (no. [%])**		
	Total^*^	**15–40**	**41–49**	***p***	**Male**	**Female**	***p***	**Saudi**	**Other**	***p***
	**(no. [%])**	**(25, 39.7%)**	**(38, 60.3%)**		**(43, 68.2%)**	**(20, 31.8%)**		**(46, 73.1%)**	**(17, 26.9%)**	
Anterior	44 (69.8%)	20 (80%)	24 (71.1%)	0.17	27 (62.8%)	17 (85%)	0.07	34 (73.9%)	10 (58.8%)	0.25
Posterior	11 (17.5%)	2 (8%)	9 (23.7%)	0.18	9 (20.9%)	2 (10%)	0.29	6 (13%)	5 (29.4%)	0.12
Ant. and post.	8 (12.7%)	3 (12%)	5 (13.2%)	0.33	7 (16.3%)	1 (5%)	0.21	6 (13%)	2 (11.8%)	0.89

*Ant., anterior; No., number; post., posterior*.

* *Numbers derived from available case analysis*.

## Discussion

Our Saudi Arabian cohort showed a distinct pattern of cerebrovascular risks factors and stroke etiologies with highest prevalence for dyslipidemia and small vessel occlusion. Although dyslipidemia has been shown to be a predominant risk factor in multiple cohorts including the large population of young stroke patients of the Helsinki Young Stroke Registry, our observation that the majority of strokes was caused by small vessel disease differs from previously reported cohorts where cervicocephalic arterial dissection and cardioembolism rank among the leading etiologies (33). This underscores the importance of comprehensive regional data on stroke cerebrovascular risk profiles and etiologies in young patients to account for geographic variance and help design targeted prevention strategies.

Subgroup analysis on age, gender, and nationality allowed a more detailed insight in cerebrovascular risk profiles and etiologies of our cohort. Both diabetes and cardiovascular disease were more prevalent in the young to middle-aged strata consistent with the results of the Helsinki Young Stroke Registry (3). On analysis of risk factors by gender, cardiovascular disease was more frequent in males than females, an observation that also shows overall agreement with previous research (3, 34). As ethnic and geographic variance in young stroke patients is well-known, we compared patients of Saudi nationality with the non-Saudi fraction of our cohort. The latter accounted for about a quarter of our study population. However, we did not observe any differences between these two groups, likely because geographic variance in phenotypes of young stroke survivors is predominantly determined by overall ethnic group rather than nationality (35). Consistently, geographic variance seems not be limited to between-country comparisons. Our data on cardiovascular risk factors differ from previous studies of stroke patients in Saudi Arabia that reported on subgroups of young stroke patients (30, 31). In these studies, hypertension was the most prevalent risk factor, whereas in our cohort, dyslipidemia, as well diabetes and smoking, was more frequent than hypertension. However, this discrepancy might be explained in parts by the inclusion of hemorrhagic stroke patients in the two aforementioned investigations as these patients are more likely suffering from hypertension than ischemic stroke patients. Notably, a recent study in a Saudi Arabian cohort analyzed the risk factors of ischemic stroke in older adults and observed risk factor patterns similar to those of the two subgroups (35).

Our results of cerebrovascular risk factor assessment in the two age strata of young stroke patients were partially consistent with the results of previous international studies of ischemic stroke in young people of similar age (9, 16–18). In an Australian study of ischemic stroke in the young, dyslipidemia, smoking, and hypertension were the most common risk factors (36). Although the study conducted analysis on two young age groups,15 to 42 years old (162 patients) and 43 to 50 years old (164 patients), the results were comparable among both groups. These observations were also similar to the results of the Helsinki Young Stroke Registry, which also analyzed two age groups, a younger group 15 to 44 years of age (*n* = 544) and an older group 45 to 49 years of age (*n* = 464) (3). Dyslipidemia was also the most common well-documented risk factor in both groups followed by smoking in the younger group, which was the third common risk factor in the older group after hypertension. By contrast, the European 15 Cities Young Stroke Study identified smoking as the most common risk factor (49%) among young stroke patients, followed by dyslipidemia (46%), which corresponds to a Greek study in young adults where smoking and dyslipidemia were the most frequent risk factors in both age groups (16, 37). In our study, diabetes was the 2nd most common risk factor, contrasting the aforementioned previous studies. The World Health Organization has reported that Saudi Arabia ranks 2nd highest in the Middle East and 7th in the world for the rate of diabetes, which might explain this discrepancy (38).

Small vessel occlusion was the most frequent etiology in our study, accounting for almost one-third of strokes, whereas ~one of five of our patients had cardioembolic causes, and 12.7% had large artery atherosclerosis. Altogether this observation is comparable with a previous study in a Jordanian population as well as retrospective analysis in a Saudi population of stroke patients (35, 39). Small vessel occlusion was found to be the most common etiology of ischemic stroke in a previous Kuwaiti study (40). By contrast, research on stroke epidemiology in Arabian Gulf countries showed that large artery atherosclerosis incidence was more frequent than small vessel occlusion (31, 41, 42). Similar to our cohort, stroke registries from Taiwan and Japan identified small vessel occlusion as the most common subtype followed by cardioembolism and large artery atherosclerosis (43, 44). Particularly in the registry from Japan, age-adjusted incidence rates in the group of ≤44 years of age were highest for small vessel occlusion, followed by large artery atherosclerosis and cardioembolism. This is also in line with our very young age strata, where small vessel occlusion constituted the most frequent stroke etiology. In an Australian study of ischemic stroke in young patients, the most frequent etiology in an older cohort aged 43 to 50 years old (*n* = 164 patients) was categorized as other determined etiology followed by cardioembolism, whereas in a younger cohort aged 15 to 42 years (*n* = 162), cardioembolism displayed the highest prevalence (36). In the Helsinki Young Stroke Registry, the most frequent etiology in the younger age group (15–44 years of age) was categorized as other determined etiology followed by undetermined etiology, whereas in the older age group (45–49 years of age), the most common etiology was small vessel occlusion (9). This observation differs from our results, in which the prevalence of cardioembolic cause was higher in our very young patients (≤40 years of age), but small vessel occlusion was most prevalent in our older strata (41–49 years of age). A case series of 394 young stroke patients in Rome yielded similar results (18). Cardioembolism was found in 34% of these patients, whereas small vessel occlusion was diagnosed in only 2.5%. A retrospective study of ischemic stroke in young adults over a period of 27 years in Spain found large artery atherosclerosis in 21% of patients, whereas cardioembolism accounted for only 17% of etiologies (17). We found a similarity between our study results and the Athens Young Stroke Registry, where small vessel occlusion (17.4%) was more frequent than cardioembolism (13.4%) in the older group (31–45 years of age, *n* = 205) (16). However, prevalence of cardioembolism was higher in the younger group (15–30 years of age, *n* = 48) with no evidence of small vessel occlusion. Our observations, viewed in conjunction with the aforementioned international research studies might suggest that in the studied Middle East and Asian regions, small vessel disease plays a more dominant role in the etiology of stroke in young individuals than in European and American populations. However, as recently pointed out in a global perspective on stroke in the young, a wide variety of possible underlying risk factors and etiologies need to be acknowledged, making it difficult to assess external validity of regional data (45).

In our cohort, the anterior circulation was by far more frequently affected (69.8%) than the posterior circulation (17.5%), whereas 12.7% of our population had a stroke in both vascular territories. This pattern is similar to previous Saudi studies results, as well-international studies of ischemic stroke in young adults (9, 19, 29, 31, 35, 46, 47). This observation was consistent among age strata in our cohort, as well as in previous populations of young stroke patients, such as the aforementioned Australian study of ischemic stroke in young patients and the Helsinki Young Stroke Registry (9, 36).

Our study is limited by its monocentric design. However, our data set derived from detailed cerebrovascular assessment and covered a 7-year observational period supporting internal validity and adding useful insight to complement the available information on the epidemiology of stroke in the young in the context of global geographic variance. While there is no clear age cutoff to discriminate stroke from stroke in the young, our observation of variable cerebrovascular phenotypes in two different age strata in a young stroke cohort highlights the need for comprehensive analysis of age-dependent cerebrovascular risk profiles and etiologies in young stroke patients. It is noteworthy that the age cutoff between pediatric and juvenile stroke displays a higher degree of agreement in the literature with individuals younger than 18 years considered pediatric. However, it remains questionable whether this age cutoff reflects age-related differences in the pathophys[i]iology of stroke. In research studies, a broader spectrum of age strata might be useful to study age-dependent differences in mechanisms of acute ischemic stroke. The knowledge deriving from this research might also help design tailored age-related strategies of diagnostic workup in young stroke patients that allow targeted assessment and avoidance of unnecessary procedures (48).

Our data support variability of patterns of cerebrovascular risk factors and stroke etiologies among regions and age groups in young stroke patients. Elaborating these differences may help formulate preventive measures by analyzing why certain populations have low prevalence in some risk factors. Nevertheless, studies that show similar distributions and frequencies of cerebrovascular risk factors underscore the need of global preventive protocols that also allow regional adaption to local epidemiological data.

## Conclusion

Cerebrovascular risk profiles and stroke etiology patterns in our cohort of young stroke patients in a Saudi Arabian cohort differed from previous studies, in particular from those outside Asia and the Middle East, underscoring the need for comprehensive global data on geographic variance. This is especially important in currently understudied regions in developing countries because detailed epidemiological information on young stroke patients forms a basis for designing individualized prevention strategies. Apparently, these strategies might benefit from an age-dependent approach as very young stroke patients display different cerebrovascular risk profiles and etiologies than young to middle-aged stroke survivors.

## Data Availability Statement

The raw data supporting the conclusions of this article will be made available by the authors, without undue reservation.

## Ethics Statement

The studies involving human participants were reviewed and approved by Institutional Review Board of King Abdullah Medical City - IRB number: 18-485. Written informed consent from the participants' legal guardian/next of kin was not required to participate in this study in accordance with the national legislation and the institutional requirements.

## Author Contributions

ME: conception, acquisition, analysis, and drafting the manuscript. AT and AS: analysis, interpretation, and revising the manuscript. EA, L-PP, JB, VP, and KB: interpretation and revising the manuscript. BI: design, interpretation, and revising the manuscript. TS: design, analysis, interpretation, and revising the manuscript. All authors contributed to the article and approved the submitted version.

## Funding

Open Access funding was enabled by the Open Access Publication Funds of the SLUB/TU Dresden.

## Conflict of Interest

TS received grants from the German Federal Ministry of Health, Kurt Goldstein Institute and German Parkinson's Association that were not related to this study and also received royalties from AstraZeneca for consulting and from Dresden International University for serving as program director and lecturer of the Master's Program in Clinical Research that were not related to this study. The remaining authors declare that the research was conducted in the absence of any commercial or financial relationships that could be construed as a potential conflict of interest.

## Publisher's Note

All claims expressed in this article are solely those of the authors and do not necessarily represent those of their affiliated organizations, or those of the publisher, the editors and the reviewers. Any product that may be evaluated in this article, or claim that may be made by its manufacturer, is not guaranteed or endorsed by the publisher.
